# Onset of periodontitis — a registry-based cohort study

**DOI:** 10.1007/s00784-023-04923-5

**Published:** 2023-02-22

**Authors:** Anna Trullenque-Eriksson, Jan Derks, Jessica Skoogh Andersson

**Affiliations:** Department of Periodontology, Institute of Odontology, The Sahlgrenska Academy at University of Gothenburg, Box 450, 405 30 Gothenburg, SE Sweden

**Keywords:** Epidemiology, Periodontitis, Risk factor(s), Periodontal status, Registries

## Abstract

**Objectives:**

The present retrospective registry-based cohort study aimed to identify parameters associated with the onset of periodontitis in young adults.

**Material and methods:**

A total of 345 Swedish subjects were clinically examined at age 19 years (as part of an epidemiological survey) and then followed up to 31 years through the Swedish Quality Registry for Caries and Periodontal diseases (SKaPa). The registry data including periodontal parameters were obtained for the period 2010–2018 (23–31 years). Logistic regression and survival models were used to identify risk factors for periodontitis (PPD ≥6 mm at ≥2 teeth).

**Results:**

The incidence of periodontitis during the 12-year observation period was 9.8%. Cigarette smoking (modified pack-years; HR 2.35, 95%CI 1.34–4.13) and increased probing pocket depth (number of sites with PPD 4–5 mm; HR 1.04, 95%CI 1.01–1.07) at 19 years were risk factors for periodontitis in subsequent young adulthood. No statistically significant association was identified for gender, snuff use, plaque and marginal bleeding scores.

**Conclusion:**

Cigarette smoking and increased probing pocket depth (≥4 mm) in late adolescence (19 years) were relevant risk factors for periodontitis in young adulthood.

**Clinical relevance:**

Our study identified cigarette smoking and increased probing depth in late adolescence as relevant risk factors of periodontitis in young adulthood. Preventive programs should therefore consider both cigarette smoking and probing pocket depths in their risk assessment.

**Supplementary Information:**

The online version contains supplementary material available at 10.1007/s00784-023-04923-5.

## Introduction

Periodontitis affects about 40% of adults [1, 2], while the more severe forms of the disease occur in ~10% of the global adult population [3, 4]. Plaque-induced gingivitis is reversible, whereas the conversion into periodontitis is characterized by a breakdown of the tooth-supporting tissues [5, 6]. Periodontitis is a multifactorial disease which is subject to individual susceptibility and has been associated with a variety of risk factors, including lifestyle-related (e.g., tobacco smoking), systemic health (e.g., diabetes), and socio-economic parameters [6–9].

Periodontitis usually has its onset after the age of 20 years and the incidence of more advanced forms peaks between the ages of 30 years and 40 years [3, 10]. While factors associated with established periodontitis have been extensively studied, the understanding of relevant risk factors in the years preceding onset is limited. To exemplify, effective self-performed oral hygiene is crucial in the prevention and treatment of periodontitis [11]. However, high levels of dental plaque and gingivitis are frequent findings in young individuals [12, 13]. To what degree these are determinants of subsequent periodontitis remains unknown. A better understanding of early risk factors may increase the effectiveness of future preventive strategies through more personalized and tailored approaches.

National health registries, such as the Swedish Quality Registry for Caries and Periodontal diseases (SKaPa), allow for longitudinal monitoring of large cohorts. In general, Swedish health registries have a high degree of completeness [14]. Thus, high external validity may be expected, which allows for reliable assessments of disease patterns. Combining an initial clinical evaluation with subsequent longitudinal registry data, the present study aims to identify risk factors for the onset of periodontitis in young adulthood.

## Material and methods

The present retrospective registry-based cohort study was approved by the Swedish Ethical Review Authority (file no: 2019-02306). The patient cohort was identified in an earlier (baseline) cross-sectional evaluation [13], for which participants provided written consent. Longitudinal follow-up was based on registry data. STROBE guidelines [15] were followed in the reporting.

### Study population

The original study population consisted of 506 subjects born in 1987 and residing in two predominantly rural counties in Sweden [for details see 13]. Briefly, individuals were examined at “age 19y” (in 2005–2006) by four calibrated registered dental hygienists. The following clinical variables were recorded (3rd molars excluded):

- Number of teeth.
- Plaque score (*Plaque Index/PI*); percentage of sites with visible plaque at four surfaces of the six Ramfjord index teeth [16].
- Marginal bleeding score (*Marginal Bleeding Index/MBI*); percentage of sites with bleeding following circumferential probing of the sulcus [17] scored at four sites per tooth.
- Probing pocket depth (PPD); registered at four sites per tooth using a UNC 15 periodontal probe. The distal surfaces of the second molars were not considered.

Marginal bone levels were measured on bitewing radiographs. The distance from the cemento-enamel junction (CEJ) to the marginal bone was assessed mesially and distally at premolars and molars, as well as at distal aspects of the canines. The number of teeth presenting with *signs of radiographic marginal bone loss* (distance CEJ to marginal bone level >2 mm) was recorded for each individual, excluding the distal surfaces of the second molars.

Participants completed a series of questionnaires regarding oral health-related perception, attitude, and behavior [18, 19]. The item “How do you consider your oral health today?” (Very good/Good/Poor/Very poor) from the Self-Perceived Oral Health questionnaire was included in the present analysis.

Information regarding tobacco habits was collected. Thus, self-reported number of cigarettes/day, snuff boxes/week and years of smoking/snuff use were noted. Individuals reporting occasional smoking (“party smoking”) were given a value of 0.5 cigarettes/day.

Considering the young age, we assumed a gradual increase in tobacco use and therefore modified the calculation of pack- and box-years at age 19 years by dividing duration by 2:

  

$$\mathrm{Modified}\;\mathrm{pack-years}=\frac{Daily\;cigarettes}{20}\times\frac{Number\;of\;years\;smoking}2$$

$$\mathrm{Modified}\;\mathrm{box-years}=\frac{Weekly\;boxes}7\times\frac{Number\;of\;years\;using\;snuff}2$$

### Data extraction and study variables

The SKaPa registry contains dental care data from individuals treated in both public and several private dental care organizations across Sweden. The registry is automatically updated from electronic dental records on a daily basis and currently includes over 7 million individuals which corresponds to >50% of the population [20, 21].

Based on the original study population (age 19 years in 2006; *N*=506) and using the unique social security identification number, a professional data analyst at SKaPa extracted registry data for the period 2010–2018 (i.e., up to 31 years of age) on an annual basis. Longitudinal data were available for 345 individuals. Baseline data for the 161 individuals for whom longitudinal data were not available from the registry are summarized in Table A1.

The mean time of follow-up (in years) was 11.0 ±1.9 (range 4–12). In all, 79.4% of the included subjects had entries in the registry representing a minimum of 5 unique years (mean 7.2 ±2.7; range 1–9).

Parameters of interest, extracted for each year of follow-up (2010–2018), included the number of teeth, number of teeth with PPD 4-5 mm, and number of teeth with PPD ≥6 mm (3rd molars excluded). An individual was considered to present with periodontitis if PPD ≥6 mm had been recorded at ≥2 teeth.

Scores on the self-rated question “How do you assess your oral health today?” (Very good/Good/Poor/Very poor), which is part of the routine examination protocol within the public dental healthcare, were also retrieved.

The registry captures legal rather than biological sex (female/male), which is hereby referred to as “gender”.

### Data analysis

Potential risk factors for the onset of periodontitis (PPD ≥6 mm at ≥2 teeth; primary outcome) were assessed through two different approaches. First, simple and multiple logistic regression models were built two evaluate the incidence of periodontitis (yes/no). In a second step, the age of onset was evaluated through survival models. For these analyses, individuals with PPD ≥6 mm or signs of radiographic marginal bone loss at baseline were excluded. All analyses were performed in Stata (Stata SE version 17.0, StataCorp LLC, TX, USA).

### Logistic regression models

Potentially relevant risk factors for the incidence of periodontitis were included in the multiple logistic regression models (function *logit*). Three separate models were built due to collinearity between risk factors (plaque index, marginal bleeding index, and sites with PPD 4–5 mm; Table A2). Results were expressed as odds ratios (ORs) with 95% confidence intervals (95%CIs).

### Survival analysis

A flexible parametric survival model (function *stpm2*) was built to illustrate the onset of periodontitis and included relevant risk factors identified in the logistic regression analyses. Cumulative incidence curves were drawn by significant risk factors. Outcomes were reported as hazard ratios (HRs) with 95%CIs.

## Results

### Status at age 19 years

Demographic, clinical, and self-assessed information from the examination at age 19 years (baseline) is provided in Table 1. A total of 56.2% of the 345 included subjects were female. Eighty-six percent of individuals considered their general health to be good, while 14% reported a variety of diseases and conditions, the most frequent being lactose intolerance (12), asthma (9), diabetes (3), and psychological disorders such as depression (3).

**Table 1:** Demographics and data from the clinical examination at age 19 years. Counts (%) and mean ±SD

***Gender***		
Female	195	(56.5%)
Male	150	(43.5%)
***Smokers*** *(N=300)*		
No	240	(80.0%)
Occasional	24	(8.0%)
Regular	36	(12.0%)
Cigarettes/day (*N*=36)	7.7	±5.0
***Years smoking*** *(N=57)*	4.0	±1.7
***Modified pack-years*** *(N=57)*	0.6	±0.7
***Snuff users*** *(N=307)*		
No	261	(85.0%)
Yes	46	(15.0%)
Snuff boxes/week (*N*=38)	2.8	±1.6
***Years using snuff*** *(N=44)*	3.4	±1.5
***Modified box-years*** *(N=37)*	0.8	±0.6
***Number of teeth***	27.3	±1.3
***Plaque Index*** (%)	45.4	±24.0
***Marginal Bleeding Index*** (%)	54.8	±18.8
***PPD ≥4 mm***		
Number of sites	6.7	±8.3
Number of teeth	4.8	±4.9
***PPD ≥6 mm***		
Number of sites	0.0	±0.1
Number of teeth	0.0	±0.1
***≥1 tooth with PPD ≥6 mm***		
No	343	(99.4%)
Yes	2	(0.6%)
***Signs of radiographic marginal bone loss***		
No	339	(98.3%)
Yes	6	(1.7%)
***Self-assessed oral health***		
Very good	120	(34.8%)
Good	195	(56.5%)
Poor	29	(8.4%)
Very poor	1	(0.3%)

*N=345 unless specified otherwise*

Thirty-six individuals were regular smokers (mean: 7.7 ±5*.*0 cigarettes/day, range 1–20), and 24 were occasional smokers. On average, subjects started smoking at age 15.0 ±1.7 years and the mean modified pack-years was 0.6 ±0.7 (range 0.03–2.5). Among the 45 snuff users (2.8 ±1.6 boxes/week, range 1–7), 5 and 9 subjects were regular and occasional smokers, respectively. Snuff users, on average, started at age 15.6 ±1.5 years and the mean modified box-years was 0.8 ±0.6 (range 0.07–2.5).

At baseline (19 years), the average number of teeth was 27.3 ±1.3, and mean PI and MBI scores were 45.4 ±24.0% and 54.8 ±18.8%, respectively. The mean number of sites with PPD ≥4 mm was 6.7 ±8.3 (4.8 ±4.9 teeth). Two individuals presented with PPD ≥6 mm (a single site in both subjects) and 6 with localized signs of radiographic marginal bone loss (1 tooth in each subject). These 8 individuals were excluded from further analyses.

Most individuals assessed their oral health to be either good or very good (91.3%).

### Status during follow-up (age 23-31y)

**Fig. 1:**
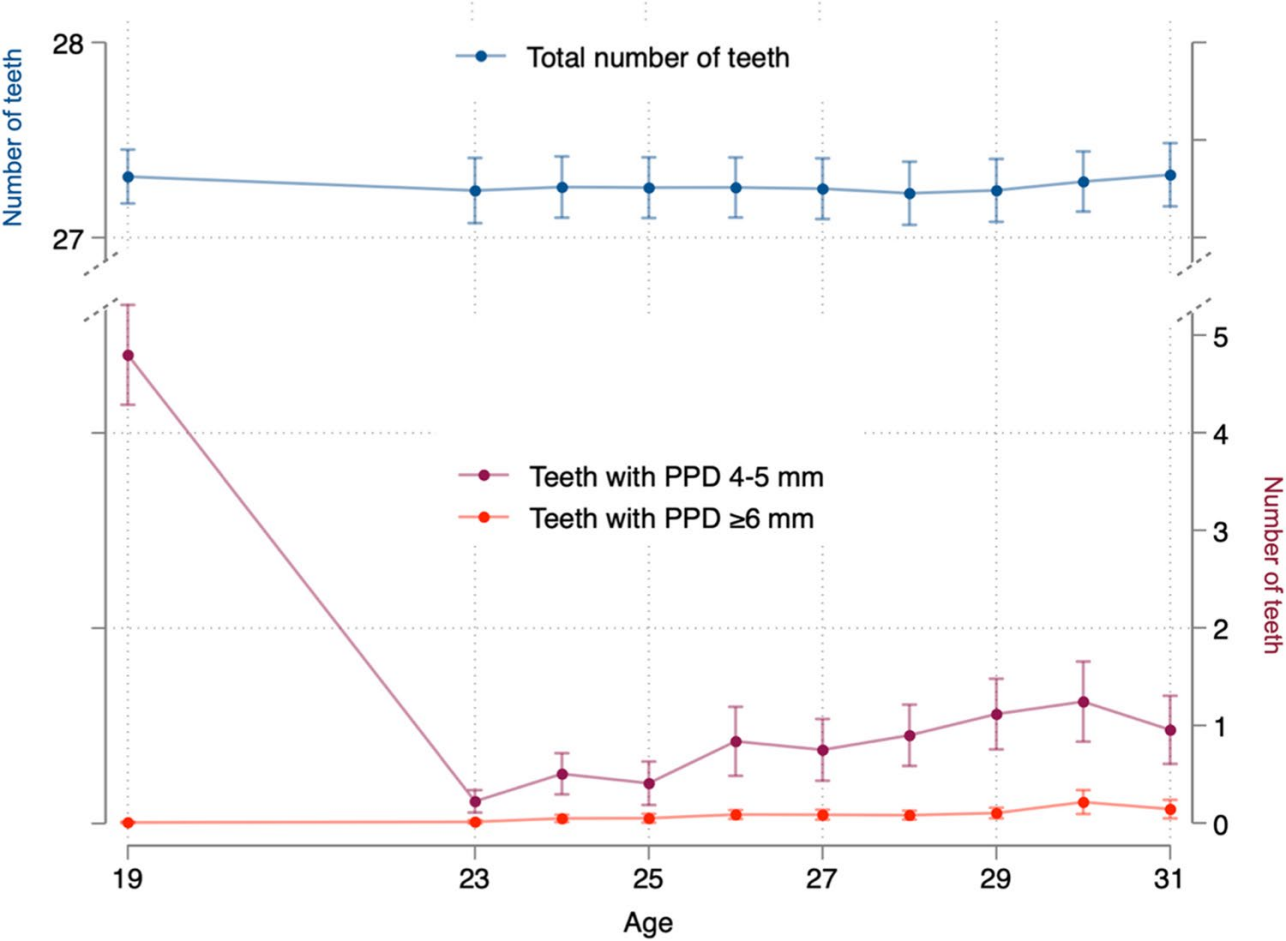
Number of teeth and number of teeth with periodontal pocketing during the follow-up period by age. *Please note that the figure has two Y-axis (for the total number of teeth to the left and pocketing to the right) and that the number of examined subjects varies at the different ages as not all participants were examined every year (see also Table A-2).*

**Fig. 2:**
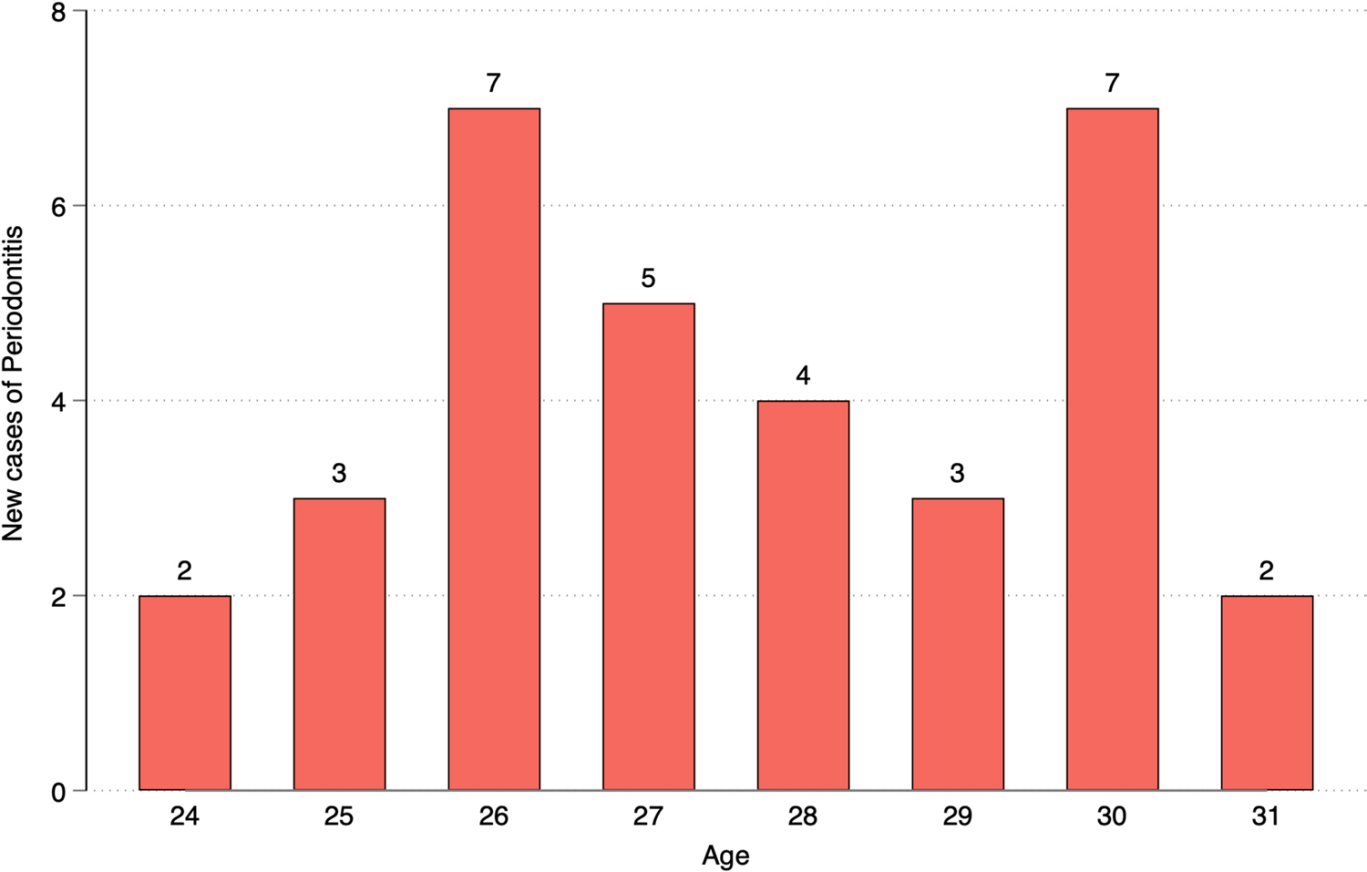
New cases of periodontitis during the follow-up period

The mean number of teeth remained unchanged over the follow-up period. The mean number of teeth with PPD ≥4 mm was lower at age 23 years and increased slightly over time (Fig. 1, Table A3). Thirty-three of the 337 subjects (9.8%) included in the analysis developed periodontitis up to age 31 years. The number of new cases by age is illustrated in Fig. 2.

**Fig. 3:**
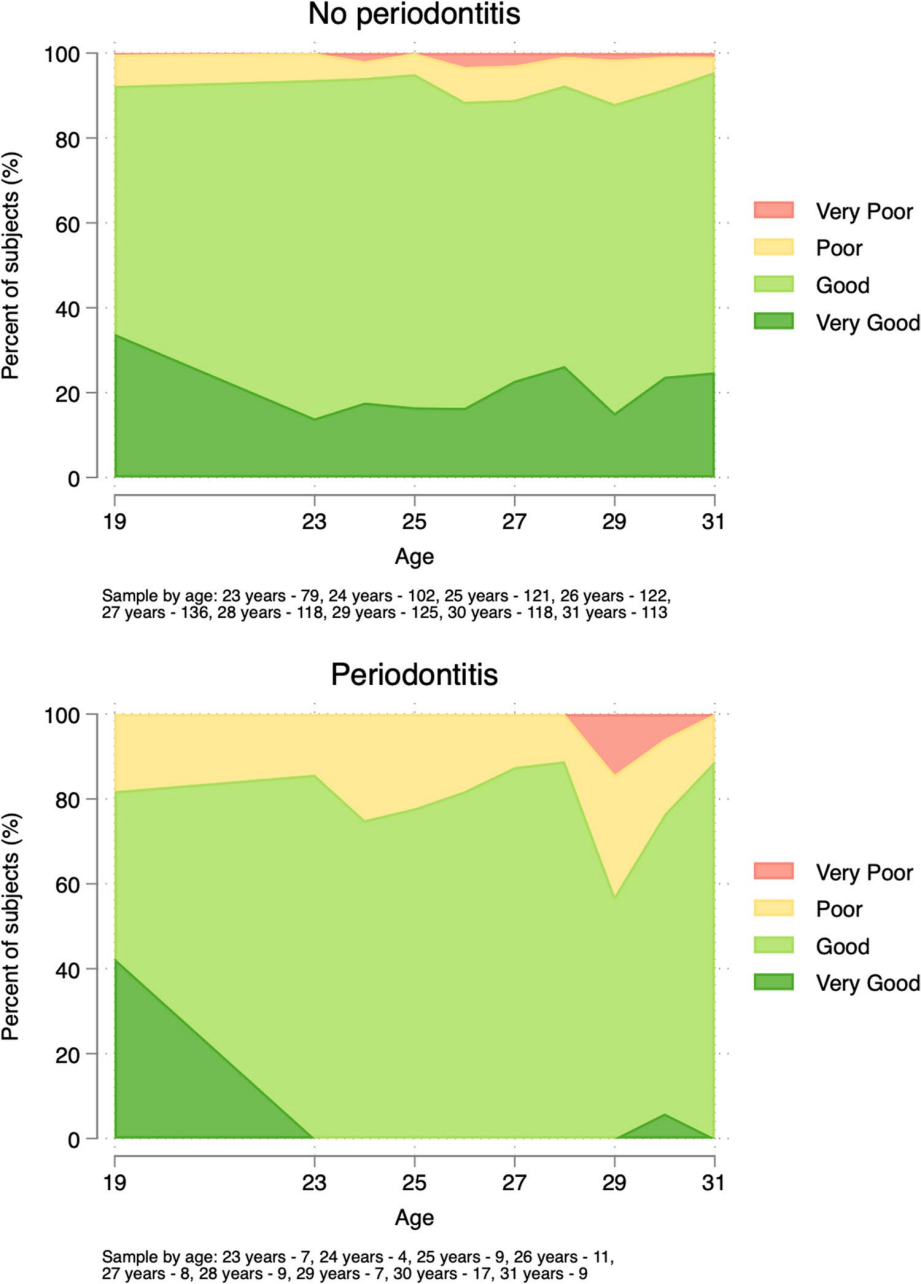
Self-assessed oral health according to the incidence of periodontitis (≥2 teeth with PPD ≥6 mm) during the follow-up period. *Please note that N varies each year, see notes below and Table A-2*

The proportion of individuals rating their oral health as impaired (poor or very poor) tended to be larger among those who developed periodontitis (Fig. 3).

### Risk factors for periodontitis

**Fig. 4.**
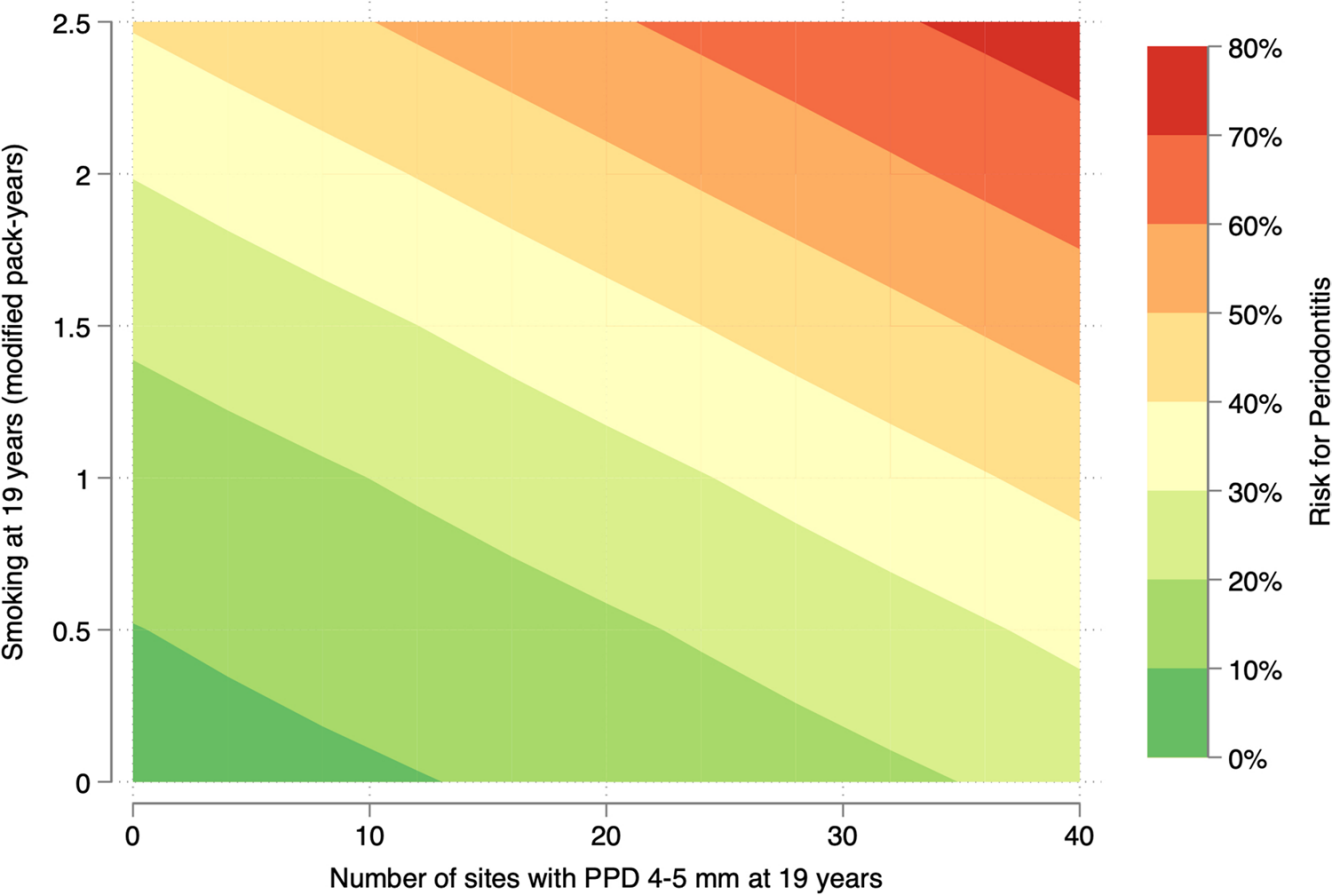
Risk for onset of periodontitis according to smoking habits at age 19 years (modified pack-years) and baseline number of sites with PPD 4–5 mm

In the simple analyses, baseline PI, number of sites/teeth with PPD 4–5 mm and smoking (dichotomized ≤10 cigarettes/day versus ≥11 cigarettes/day, and modified pack-years) were associated with periodontitis, whereas no association with gender, snuff use (dichotomized yes/no and modified box-years) or MBI was noted (see Tables 2 and A4). In the adjusted models, the association remained significant for smoking (modified pack-years; adjusted OR 2.56, 95%CI 1.26–5.20) and the number of sites with PPD 4–5 mm (adjusted OR 1.04, 95%CI 1.00–1.08) (Fig. 4; Tables 2 and A5).

**Table 2:** Logistic regression analyses for the onset of periodontitis (≥2 teeth with PPD ≥6 mm; dependent variable)

Independent variables	Unadjusted OR (95%CI)	Adjusted model 1^‡^ OR (95%CI)	Adjusted model 2^‡^ OR (95%CI)	Adjusted model 3^‡^ OR (95%CI)
**Gender** *(ref: female)* Male	1.94 (0.94–4.02) *p=0.073*	1.97 (0.85–4.54) *p=0.113*	2.08 (0.92–4.71) *p=0.080*	1.96 (0.86–4.45) *p=0.109*
**Smoking**^†^ *(ref: ≤10 cigarettes/day)* ≥11 cigarettes/day	7.50* (1.59–35.35) *p=0.011*	-	-	-
**Modified pack-years**^‡^ (continuous)	2.60* (1.34–5.04) *p=0.005*	2.52* (1.24–5.13) *p=0.010*	2.67* (1.33–5.34) *p=0.006*	2.56* (1.26–5.20) *p=0.009*
**Snuff use**^*§*^ *(ref: no)* Yes	1.65 (0.63–4.32) *p=0.308*	-	-	-
**Modified box-years**^¶^ (continuous)	0.72 (0.18 – 2.99) *p=0.655*	-	-	-
**Plaque index** (continuous)	1.02* (1.00–1.03) *p=0.018*	1.01 (0.99–1.03) *p=0.240*	-	-
**Marginal bleeding index** (continuous)	1.02 (1.00–1.04) *p=0.103*	-	1.01 (0.99–1.04) *p=0.282*	-
**Sites with PPD 4–5 mm** (continuous)	1.05* (1.01–1.08) *p=0.009*	-	**-**	1.04* (1.00–1.08) *p=0.047*
**Teeth with PPD 4–5 mm** (continuous)	1.08* (1.01–1.15) *p=0.016*	-	-	-

*Subjects with PPD ≥6 mm or signs of radiographic marginal bone loss at baseline have been excluded*

*Adjusted model 1 includes gender, modified pack-years, plaque index*

*Adjusted model 2 includes gender, modified pack-years, marginal bleeding index*

*Adjusted model 3 includes gender, modified pack-years, sites with PPD 4–5 mm*

*N=337 (except for* ^†^*N=293,* ^‡^*N=290,* ^*§*^*N=300,* ^**¶**^*N=292)*

*p<0.05

**Fig. 5:**
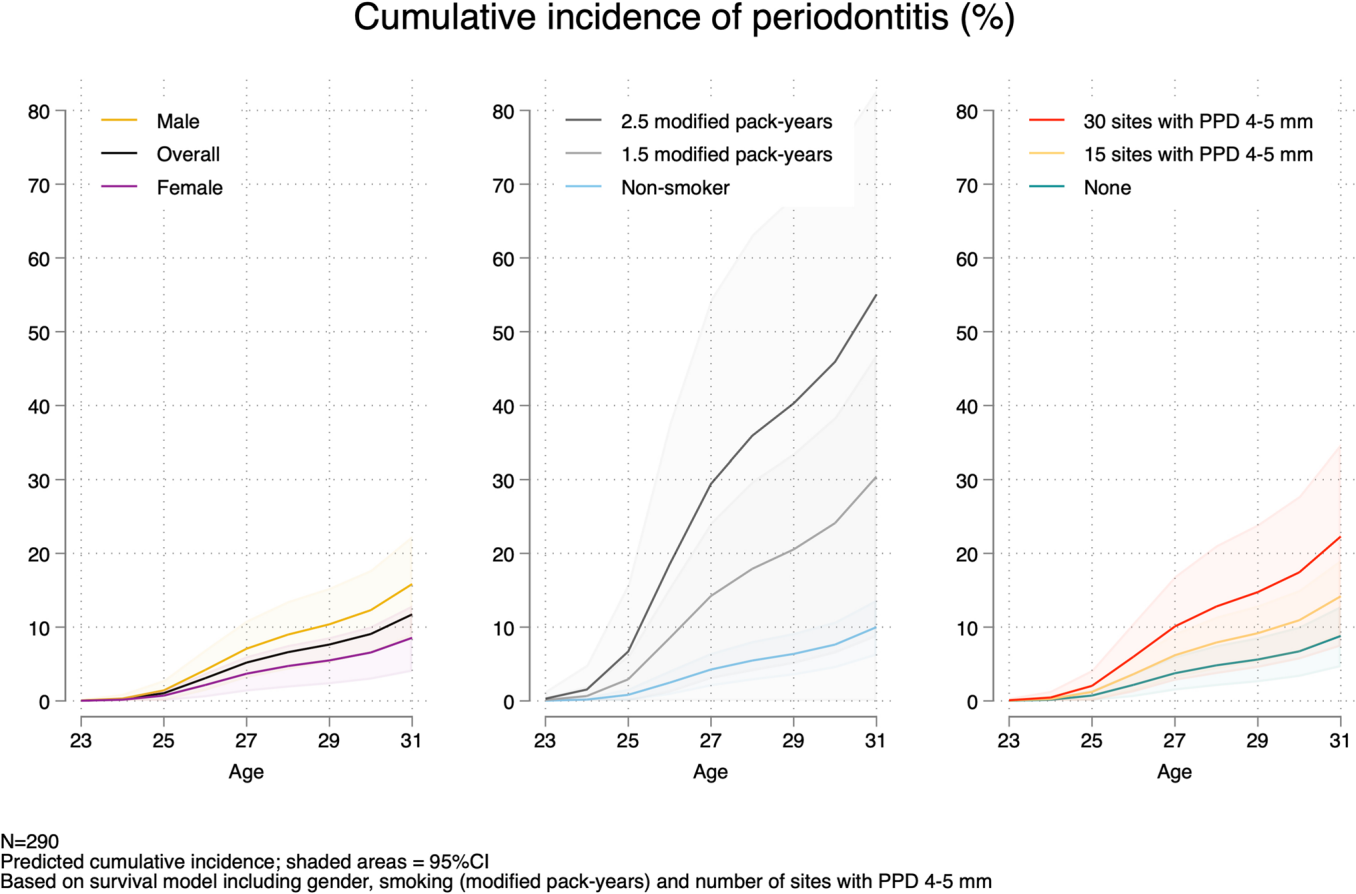
Cumulative incidence of periodontitis (≥2 teeth with PPD ≥6 mm) by risk factors included in the survival model

Figure 5 illustrates the cumulative incidence of periodontitis by potential risk factors included in adjusted model 3. In the survival model, both modified pack-years at 19 years (HR 2.35, 95%CI 1.34–4.13) and baseline number of sites with PPD 4–5 mm (HR 1.04, 95%CI 1.01–1.07) were significant risk factors for future periodontitis (Table A6). The association with gender was not statistically significant (*p*=0.074).

## Discussion

The purpose of the present study was to identify parameters associated with the onset of periodontitis in young adults. Up to 31 years of age, the incidence of periodontitis was 9.8%. Cigarette smoking and increased probing pocket depth (≥4 mm) in late adolescence (19 years) were risk factors for the onset of periodontitis in subsequent young adulthood (modified pack-years HR 2.35; number of sites with PPD 4–5 mm HR 1.04). No significant association was identified for gender, snuff use, plaque or marginal bleeding scores.

The incidence of periodontitis in the present study was assessed based solely on the presence of deep periodontal probing (at least 2 teeth displaying PPD ≥6 mm), as the SKaPa registry lacks reliable data on attachment loss. Thus, our case definition does not fully adhere to the recommendations proposed by the 2017 World Workshop [7]. It should be noted, however, that epidemiological studies have used a variety of definitions for periodontitis [3, 4]. Similar to our methodology, the Community Periodontal Index of Treatment Needs (CPITN), for instance, relies on probing pocket depth as the single criterion to identify cases of periodontitis. In other epidemiological studies such as NHANES [e.g., 2] and SHIP [e.g., 22], clinical attachment levels were considered. The longitudinal evaluations from Jönköping also assessed marginal bone levels [e.g., 1]. This issue reflects one limitation of registry research, where, as in our case, detailed information on clinical attachment levels, bleeding on probing and/or marginal bone levels was not available.

Nevertheless, the incidence of periodontitis of just below 10% corresponds well with the concept that the age span of 20–30 years may represent a critical period in the development of the disease. Thorbert-Mros et al. [10], for instance, evaluated disease onset retrospectively in patients presenting with generalized severe periodontitis. Using radiographic data, the authors identified ages 22 to 28 years to be a typical period during which initial signs of periodontal attachment loss become evident. Evidence points to a peak incidence of severe periodontitis in the late thirties [3, 23]. Corresponding estimates for advanced forms of disease were not possible as the present patient cohort was still in their mid-thirties.

Cigarette smoking was the most relevant risk factor for periodontitis in the present study, with a clear dose-dependent effect. Tobacco smoking has previously been identified as an important factor in the onset and progression of periodontitis, both in early adulthood [24] and later in life [25, 26]. Smoking was also considered a criterion for prognosis-grading in the most recent classification of periodontitis [7]. The lack of association between snuff use and periodontitis is in line with previous data [27, 28].

We described tobacco use in late adolescence through a modification of the pack/box-years measure. The duration of smoking/snuff use was given a smaller relative importance assuming that tobacco dosage gradually increased during adolescence. We relied solely on self-reported data and could not confirm whether smokers and snuff users continued the habit into young adulthood. These are limitations that must be considered when interpreting the results.

The present study evaluated marginal bleeding at age 19 years rather than bleeding on probing. It could be argued that in individuals not presenting with deep pocketing, differences between the two measures are probably negligible. While the number of sites with PPD ≥4 mm in late adolescence (age 19 years) was a relevant risk factor for periodontitis, gingival bleeding was not. The latter may seem in conflict with data from long-term follow-up of periodontally susceptible individuals, where bleeding scores were generally attributed to high predictive values for disease progression [29–31]. The population was markedly different from those referenced above, both in terms of age and most likely also in terms of disease susceptibility. Furthermore, gingival inflammation has been shown to be highly prevalent in younger ages [12, 32, 33], which may limit its prognostic value. Thus, parameters other than bleeding on probing may be more suitable for prognostic models in younger age groups. The decline in the number of teeth with PPD ≥4 mm from 19 years (4.8) to the later follow-up (23–31 years; 0.2–1.7) is not fully understood but may be related to improved oral hygiene habits in early adult life.

Previous studies reported a higher prevalence of periodontitis in males [2, 22]. While the incidence in the present study tended to be greater in males, differences by gender did not reach statistical significance. The discrepancy between our data and the results described elsewhere might be explained by the obvious differences in age but also by generational aspects.

Interestingly, a considerable proportion of individuals who developed periodontitis (>60%) still rated their oral health to be good. Considering the important role of the patient in the prevention and treatment of periodontitis, the apparent lack of disease awareness is noteworthy. Preventive efforts may become more effective if they are combined with further emphasis on patient education.

There are some further limitations that should be considered when interpreting the results from this study and its external validity. Our sample consists of subjects in Sweden seeking dental care from clinicians providing data to SKaPa, which may have entailed a selection bias. Furthermore, it was not possible to assess whether non-attenders (32% of the initial sample) differed in disease incidence. Also, the statistical power in detecting relevant risk factors, for instance, gender, may have been limited.

## Conclusion

The incidence of periodontitis was not an uncommon event in young adults. Cigarette smoking and increased probing pocket depth (≥4 mm) in late adolescence (19 years) were relevant risk factors for periodontitis in young adulthood.

## Supplementary information

Supplementary materials 1: Table A1. Baseline data for 161 individuals for whom longitudinal data was not available. *Counts (%) and mean ±SD.* Table A2. Pairwise correlations (P-value). Table A3. Data from SKaPa registry (2010-2018). Table A4. Logistic regression (unadjusted; dependent variable: onset of periodontitis) (N=337 unless specified otherwise). Table A5. Logistic regression (adjusted for gender and modified pack-years; dependent variable: onset of periodontitis) (N=290). Table A6. Survival analysis for Periodontitis (≥2 teeth with PPD ≥6 mm; adjusted for gender, modified pack-years and number of sites with PPD 4-5 mm) (N=290). (DOCX 39.1 KB)

## Data Availability

The data that support the findings of this study are available from the authors upon reasonable request.

## References

[1] Wahlin A, Papias A, Jansson H, Norderyd O (2018). Secular trends over 40 years of periodontal health and disease in individuals aged 20-80 years in Jonkoping, Sweden: repeated cross-sectional studies. J Clin Periodontol.

[2] Eke PI (2015). Update on prevalence of periodontitis in adults in the United States: NHANES 2009 to 2012. J Periodontol.

[3] Kassebaum NJ, Bernabe E, Dahiya M, Bhandari B, Murray CJ, Marcenes W (2014). Global burden of severe periodontitis in 1990-2010: a systematic review and meta-regression. J Dent Res.

[4] Chen MX, Zhong YJ, Dong QQ, Wong HM, Wen YF (2021). Global, regional, and national burden of severe periodontitis, 1990-2019: an analysis of the Global Burden of Disease Study 2019. J Clin Periodontol.

[5] Murakami S, Mealey BL, Mariotti A, Chapple ILC (2018). Dental plaque-induced gingival conditions. J Periodontol.

[6] Tonetti MS, Greenwell H, Kornman KS (2018). Staging and grading of periodontitis: framework and proposal of a new classification and case definition. J Periodontol.

[7] Papapanou PN (2018). Periodontitis: consensus report of workgroup 2 of the 2017 World Workshop on the Classification of Periodontal and Peri-Implant Diseases and Conditions. J Clin Periodontol.

[8] Holde GE, Baker SR, Jonsson B (2018). Periodontitis and quality of life: what is the role of socioeconomic status, sense of coherence, dental service use and oral health practices? An exploratory theory-guided analysis on a Norwegian population. J Clin Periodontol.

[9] Kongstad J, Enevold C, Christensen LB, Fiehn NE, Holmstrup P (2017). Impact of periodontitis case criteria: a cross-sectional study of lifestyle. J Periodontol.

[10] Thorbert-Mros S, Cassel B, Berglundh T (2017). Age of onset of disease in subjects with severe periodontitis: A 9- to 34-year retrospective study. J Clin Periodontol.

[11] Axelsson P, Nystrom B, Lindhe J (2004). The long-term effect of a plaque control program on tooth mortality, caries and periodontal disease in adults. Results after 30 years of maintenance. J Clin Periodontol.

[12] Abrahamsson KH, Koch G, Norderyd O, Romao C, Wennstrom JL (2006). Periodontal conditions in a Swedish city population of adolescents: a cross-sectional study. Swed Dent J.

[13] Ericsson JS, Abrahamsson KH, Ostberg AL, Hellstrom MK, Jonsson K, Wennström JL (2009). Periodontal health status in Swedish adolescents: an epidemiological, cross-sectional study. Swed Dent J.

[14] Emilsson L, Lindahl B, Köster M, Lambe M, Ludvigsson JF (2015). Review of 103 Swedish Healthcare Quality Registries. J. Intern. Med..

[15] von Elm E (2007). The strengthening the reporting of observational studies in epidemiology (STROBE) statement: guidelines for reporting observational studies. Lancet.

[16] Ramfjord SP (1967). The periodontal disease index (PDI). J Periodontol.

[17] Ainamo J, Bay I (1975). Problems and proposals for recording gingivitis and plaque. Int Dent J.

[18] Östberg AL (2002). On self-perceived oral health in Swedish adolescents. Swed Dent J.

[19] Ericsson JS, Östberg AL, Wennström JL, Abrahamsson KH (2012). Oral health-related perceptions, attitudes, and behavior in relation to oral hygiene conditions in an adolescent population. Eur J Oral Sci.

[20] von Bültzingslöwen I, Östholm H, Gahnberg L, Ericson D, Wennström JL, Paulander J (2019). Swedish Quality Registry for Caries and Periodontal Diseases - a framework for quality development in dentistry. Int Dent J.

[21] SKaPa. "Årsrappport 2020." https://www.skapareg.se/wp-content/uploads/2021/06/SKaPa_2020-årsrapport.pdf. Accessed 29 Sept 2022.

[22] Gätke D, Holtfreter B, Biffar R, Kocher T (2012). Five-year change of periodontal diseases in the Study of Health in Pomerania (SHIP). J Clin Periodontol.

[23] Thomson WM, Shearer DM, Broadbent JM, Foster Page LA, Poulton R (2013). The natural history of periodontal attachment loss during the third and fourth decades of life. J Clin Periodontol.

[24] Thomson WM, Broadbent JM, Welch D, Beck JD, Poulton R (2007). Cigarette smoking and periodontal disease among 32-year-olds: a prospective study of a representative birth cohort. J Clin Periodontol.

[25] Bergström J, Eliasson S, Dock J (2000). Exposure to tobacco smoking and periodontal health. J Clin Periodontol.

[26] Ravida A (2020). Dose-dependent effect of smoking and smoking cessation on periodontitis-related tooth loss during 10 - 47 years periodontal maintenance-a retrospective study in compliant cohort. J Clin Periodontol.

[27] Bergström J, Keilani H, Lundholm C, Rådestad U (2006). Smokeless tobacco (snuff) use and periodontal bone loss. J Clin Periodontol.

[28] Hugoson A, Rolandsson M (2011). Periodontal disease in relation to smoking and the use of Swedish snus: epidemiological studies covering 20 years (1983-2003). J Clin Periodontol.

[29] Lang NP, Joss A, Orsanic T, Gusberti FA, Siegrist BE (1986). Bleeding on probing. A predictor for the progression of periodontal disease?. J Clin Periodontol.

[30] Lang NP, Nyman S, Senn C, Joss A (1991). Bleeding on probing as it relates to probing pressure and gingival health. J Clin Periodontol.

[31] Matuliene G (2008). Influence of residual pockets on progression of periodontitis and tooth loss: results after 11 years of maintenance. J Clin Periodontol.

[32] Hugoson A, Norderyd O, Slotte C, Thorstensson H (1998). Oral hygiene and gingivitis in a Swedish adult population 1973, 1983 and 1993. J Clin Periodontol.

[33] Botero JE, Rosing CK, Duque A, Jaramillo A, Contreras A (2015). Periodontal disease in children and adolescents of Latin America. Periodontol. 2000.

