# Supplementary material for: Onset of periodontitis — a registry-based cohort study
Source: Clin Oral Investig. 2023 Feb 22;27(5):2187–95. doi: 10.1007/s00784-023-04923-5 (PMC10160190; doi:10.1007/s00784-023-04923-5)
Supplement: Supplementary file 1 — Table A1. Baseline data for 161 individuals for whom longitudinal data was not available. Counts (%) and mean ±SD. Table A2. Pairwise correlations (P-value). Table A3. Data from SKaPa registry (2010-2018). Table A4. Logistic regression (unadjusted; dependent variable: onset of periodontitis) (N=337 unless specified otherwise). Table A5. Logistic regression (adjusted for gender and modified pack-years; dependent variable: onset of periodontitis) (N=290). Table A6. Survival analysis for Periodontitis (≥2 teeth with PPD ≥6 mm; adjusted for gender, modified pack-years and number of sites with PPD 4-5 mm) (N=290). (DOCX 39.1 KB) [file 784_2023_4923_MOESM1_ESM.docx]

STROBE Statement—Checklist of items that should be included in reports of ***cohort studies***

|  | Item No | Recommendation |  |
| --- | --- | --- | --- |
| **Title and abstract** | 1 | (*a*) Indicate the study’s design with a commonly used term in the title or the abstract | Title and First paragraph in Abstract |
|  |  | (*b*) Provide in the abstract an informative and balanced summary of what was done and what was found | Second and third paragraph in Abstract |
| Introduction | | |  |
| Background/rationale | 2 | Explain the scientific background and rationale for the investigation being reported | First, second & third paragraph in Introduction |
| Objectives | 3 | State specific objectives, including any prespecified hypotheses | Last paragraph in Introduction |
| Methods | | |  |
| Study design | 4 | Present key elements of study design early in the paper | First paragraph in Material and Methods |
| Setting | 5 | Describe the setting, locations, and relevant dates, including periods of recruitment, exposure, follow-up, and data collection | “Study population” and  “Data extraction and study variables” in Material and Methods |
| Participants | 6 | (*a*) Give the eligibility criteria, and the sources and methods of selection of participants. Describe methods of follow-up | “Study population” and  “Data extraction and study variables” in Material and Methods |
|  |  | (*b*) For matched studies, give matching criteria and number of exposed and unexposed | - |
| Variables | 7 | Clearly define all outcomes, exposures, predictors, potential confounders, and effect modifiers. Give diagnostic criteria, if applicable | “Study population”,  “Data extraction and study variables” and “Data analysis” in Material and Methods |
| Data sources/ measurement | 8* | For each variable of interest, give sources of data and details of methods of assessment (measurement). Describe comparability of assessment methods if there is more than one group | “Study population” and  “Data extraction and study variables” in Material and Methods |
| Bias | 9 | Describe any efforts to address potential sources of bias | - |
| Study size | 10 | Explain how the study size was arrived at | “Study population” and  “Data extraction and study variables” in Material and Methods |
| Quantitative variables | 11 | Explain how quantitative variables were handled in the analyses. If applicable, describe which groupings were chosen and why | “Data analysis” in Material and Methods |
| Statistical methods | 12 | (*a*) Describe all statistical methods, including those used to control for confounding | “Data analysis” in Material and Methods |
|  |  | (*b*) Describe any methods used to examine subgroups and interactions | “Data analysis” in Material and Methods |
|  |  | (*c*) Explain how missing data were addressed | “Data analysis” in Material and Methods |
|  |  | (*d*) If applicable, explain how loss to follow-up was addressed | - |
|  |  | (*e*) Describe any sensitivity analyses | - |
| Results | | |  |
| Participants | 13* | (a) Report numbers of individuals at each stage of study—eg numbers potentially eligible, examined for eligibility, confirmed eligible, included in the study, completing follow-up, and analysed | “Study population” and  “Data extraction and study variables” in Material and Methods as well as “Status at age 19y” and “Status during follow-up (age 23-31 years)” in Results; “N” reported in tables & figures |
|  |  | (b) Give reasons for non-participation at each stage | - |
|  |  | (c) Consider use of a flow diagram | - |
| Descriptive data | 14* | (a) Give characteristics of study participants (eg demographic, clinical, social) and information on exposures and potential confounders | “Status at age 19y” and “Status during follow-up (age 23-31 years)” in Results; Table 1 |
|  |  | (b) Indicate number of participants with missing data for each variable of interest | “N” reported in tables & figures |
|  |  | (c) Summarise follow-up time (eg, average and total amount) | “Study population” and  “Data extraction and study variables” in Material and Methods as well as “Status at age 19y” and “Status during follow-up (age 23-31 years)” in Results |
| Outcome data | 15* | Report numbers of outcome events or summary measures over time | “Status at age 19y” and “Status during follow-up (age 23-31 years)” in Results; Table A-2; Figure 1,2,3 |
| Main results | 16 | (*a*) Give unadjusted estimates and, if applicable, confounder-adjusted estimates and their precision (eg, 95% confidence interval). Make clear which confounders were adjusted for and why they were included | “Status at age 19y”, “Status during follow-up (age 23-31 years)” and “Risk factors for periodontitis” in Results; Tables 2, A-1, A-3, A-4, A-5; Figure 4,5 |
|  |  | (*b*) Report category boundaries when continuous variables were categorized | “Data extraction and study variables” in Material and Methods |
|  |  | (*c*) If relevant, consider translating estimates of relative risk into absolute risk for a meaningful time period | - |
| Other analyses | 17 | Report other analyses done—eg analyses of subgroups and interactions, and sensitivity analyses | - |
| Discussion | | |  |
| Key results | 18 | Summarise key results with reference to study objectives | First paragraph in Discussion |
| Limitations | 19 | Discuss limitations of the study, taking into account sources of potential bias or imprecision. Discuss both direction and magnitude of any potential bias | Paragraphs 2, 3, 5, 9 in Discussion |
| Interpretation | 20 | Give a cautious overall interpretation of results considering objectives, limitations, multiplicity of analyses, results from similar studies, and other relevant evidence | Second to nineth paragraph in Discussion |
| Generalisability | 21 | Discuss the generalisability (external validity) of the study results | Last paragraph in Discussion |
| Other information | | |  |
| Funding | 22 | Give the source of funding and the role of the funders for the present study and, if applicable, for the original study on which the present article is based | Funding statement |

*Give information separately for exposed and unexposed groups.

**Note:** An Explanation and Elaboration article discusses each checklist item and gives methodological background and published examples of transparent reporting. The STROBE checklist is best used in conjunction with this article (freely available on the Web sites of PLoS Medicine at http://www.plosmedicine.org/, Annals of Internal Medicine at http://www.annals.org/, and Epidemiology at http://www.epidem.com/). Information on the STROBE Initiative is available at http://www.strobe-statement.org.

| Table A1. Baseline data for 161 individuals for whom longitudinal data was not available. *Counts (%) and mean ±SD* | | |
| --- | --- | --- |
| ***Gender^a^*** |  | |
| Female | 76 | (47.2%) |
| Male | 85 | (52.8%) |
| ***Smokers*** *(N=140)* |  |  |
| No | 115 | (82.1%) |
| Occasional | 7 | (5.0%) |
| Regular | 18 | (12.9%) |
| Cigarettes/day (N=17) | 9.0 | ±5.4 |
| ***Snuff users*** *(N=144)* ***^b^*** |  |  |
| No | 110 | (76.4%) |
| Yes | 34 | (23.6%) |
| Snuff boxes/week (N=29) | 2.5 | ±1.3 |
| ***Number of teeth*** | 27.1 | ±1.5 |
| ***Plaque Index*** (%) | 49.3 | ±23.6 |
| ***Marginal Bleeding Index*** (%) | 57.2 | ±19.8 |
| ***PPD ≥4 mm*** |  |  |
| Number of sites | 8.1 | ±9.5 |
| Number of teeth | 5.6 | ±5.4 |
| ***PPD ≥6 mm*** |  |  |
| Number of sites***^c^*** | 0.1 | ±0.4 |
| Number of teeth***^d^*** | 0.1 | ±0.4 |
| ***≥1 tooth with PPD ≥6 mm*** |  |  |
| No | 155 | (96.3%) |
| Yes | 6 | (3.7%) |
| ***Signs of radiographic marginal bone loss*** |  |  |
| No | 158 | (98.1%) |
| Yes | 3 | (1.9%) |
| ***Self-assessed oral health*** (N=160) |  |  |
| Very good | 52 | (32.5%) |
| Good | 87 | (54.4%) |
| Poor | 21 | (13.1%) |
| Very poor | 0 |  |

| *N=161 unless specified otherwise*  *^a^ Statistically significant difference compared to included individuals (chi2 p=0.044)*  *^b^ Statistically significant difference compared to included individua (chi2 p=0.020)*  *^c^ Statistically significant difference compared to included individua (t-test p=0.006)*  *^d^ Statistically significant difference compared to included individua (t-test p=0.013)* |
| --- |

**Table A2. Pairwise correlations** (P-value)

| **Variables** | **Baseline Plaque Index** | **Baseline Marginal bleeding Index** | **Baseline sites with PPD 4-5 mm** | **Baseline teeth with PPD 4-5 mm** |
| --- | --- | --- | --- | --- |
| Baseline Plaque Index | 1.000 |  |  |  |
|  |  |  |  |  |
| Baseline Marginal Bleeding Index | **0.619*** | 1.000 |  |  |
|  | (<0.001) |  |  |  |
| Baseline sites with PPD 4-5 mm | **0.384*** | **0.555*** | 1.000 |  |
|  | (<0.001) | (<0.001) |  |  |
| Baseline teeth with PPD 4-5 mm | **0.426*** | **0.589*** | **0.962*** | 1.000 |
|  | (<0.001) | (<0.001) | (<0.001) |  |

** p<0.05*

*N=345*

| **Table A3. Data from SKaPa registry (2010-2018)**   \|  \| **Age (years)** \| \| \| \| \| \| \| \| \| \| \| \| \| \| \| \| \| \| \| \| \| --- \| --- \| --- \| --- \| --- \| --- \| --- \| --- \| --- \| --- \| --- \| --- \| --- \| --- \| --- \| --- \| --- \| --- \| --- \| --- \| --- \| \|  \| **19** \| \| **23** \| \| **24** \| \| **25** \| \| **26** \| \| **27** \| \| **28** \| \| **29** \| \| **30** \| \| **31** \| \| \|  \| (N=345)  *Mean (SD)* \| \| (N=257)  *Mean (SD)* \| \| (N=278)  *Mean (SD)* \| \| (N=285)  *Mean (SD)* \| \| (N=288)  *Mean (SD)* \| \| (N=283)  *Mean (SD)* \| \| (N=286)  *Mean (SD)* \| \| (N=285)  *Mean (SD)* \| \| (N=275)  *Mean (SD)* \| \| (N=239)  *Mean (SD)* \| \| \| ***Number of teeth*** \| 27.3 \| (1.3) \| 27.2 \| (1.4) \| 27.3 \| (1.3) \| 27.3 \| (1.3) \| 27.3 \| (1.3) \| 27.3 \| (1.3) \| 27.2 \| (1.4) \| 27.2 \| (1.4) \| 27.3 \| (1.3) \| 27.3 \| (1.3) \| \| ***Number of teeth with PPD ≥4 mm*** \| 4.8 \| (4.8) \| 0.2 \| (0.9) \| 0.5 \| (1.8) \| 0.4 \| (1.9) \| 0.8 \| (3.1) \| 0.7 \| (2.7) \| 0.9 \| (2.7) \| 1.1 \| (3.1) \| 1.2 \| (3.5) \| 1.0 \| (2.8) \| \| ***Number of teeth with PPD 4-5 mm*** \| 4.8 \| (4.9) \| 0.2 \| (1.0) \| 0.6 \| (1.9) \| 0.5 \| (2.1) \| 0.9 \| (3.3) \| 0.8 \| (3.0) \| 1.0 \| (2.9) \| 1.2 \| (3.4) \| 1.5 \| (3.9) \| 1.1 \| (3.1) \| \| ***Number of teeth with PPD ≥6 mm*** \| 0.0 \| (0.1) \| 0.0 \| (0.1) \| 0.0 \| (0.3) \| 0.0 \| (0.4) \| 0.1 \| (0.4) \| 0.1 \| (0.4) \| 0.1 \| (0.4) \| 0.1 \| (0.5) \| 0.2 \| (1.0) \| 0.1 \| (0.7) \| \| ***Self-assessed oral health*** \| (N=345)  *Count (%)* \| \| (N=87)  *Count (%)* \| \| (N=107)  *Count (%)* \| \| (N=132)  *Count (%)* \| \| (N=135)  *Count (%)* \| \| (N=144)  *Count (%)* \| \| (N=128)  *Count (%)* \| \| (N=133)  *Count (%)* \| \| (N=135)  *Count (%)* \| \| (N=123)  *Count (%)* \| \| \| Very good \| 120 \| (34.8) \| 11 \| (12.6) \| 18 \| (16.8) \| 20 \| (15.2) \| 20 \| (14.8) \| 31 \| (21.5) \| 31 \| (24.2) \| 19 \| (14.3) \| 29 \| (21.5) \| 28 \| (22.8) \| \| Good \| 195 \| (56.5) \| 70 \| (80.5) \| 82 \| (76.6) \| 104 \| (78.8) \| 99 \| (73.3) \| 97 \| (67.4) \| 87 \| (68.0) \| 96 \| (72.2) \| 92 \| (68.1) \| 89 \| (72.4) \| \| Poor \| 29 \| (8.4) \| 6 \| (6.9) \| 5 \| (4.7) \| 8 \| (6.1) \| 12 \| (8.9) \| 12 \| (8.3) \| 9 \| (7.0) \| 15 \| (11.3) \| 12 \| (8.9) \| 5 \| (4.1) \| \| Very poor \| 1 \| (0.3) \| 0 \| (0.0) \| 2 \| (1.9) \| 0 \| (0.0) \| 4 \| (3.0) \| 4 \| (2.8) \| 1 \| (0.8) \| 3 \| (2.3) \| 2 \| (1.5) \| 1 \| (0.8) \| \| ***Periodontitis*** \| (N=345)  *Count (%)* \| \| (N=257)  *Count (%)* \| \| (N=278)  *Count (%)* \| \| (N=285)  *Count (%)* \| \| (N=288)  *Count (%)* \| \| (N=283)  *Count (%)* \| \| (N=286)  *Count (%)* \| \| (N=285)  *Count (%)* \| \| (N=275)  *Count (%)* \| \| (N=239)  *Count (%)* \| \| \| No \| 345 \| (100.0) \| 256 \| (99.6) \| 275 \| (98.9) \| 282 \| (98.9) \| 279 \| (96.9) \| 274 \| (96.8) \| 279 \| (97.6) \| 276 \| (96.8) \| 262 \| (95.3) \| 232 \| (97.1) \| \| Yes \| 0 \| (0.0) \| 1 \| (0.4) \| 3 \| (1.1) \| 3 \| (1.1) \| 9 \| (3.1) \| 9 \| (3.2) \| 7 \| (2.4) \| 9 \| (3.2) \| 13 \| (4.7) \| 7 \| (2.9) \| \|  \|  \|  \|  \|  \|  \|  \|  \|  \|  \|  \|  \|  \|  \|  \|  \|  \|  \|  \|  \|  \| |
| --- | --- | --- | --- | --- | --- | --- | --- | --- | --- | --- | --- | --- | --- | --- | --- | --- | --- | --- | --- | --- | --- | --- | --- | --- | --- | --- | --- | --- | --- | --- | --- | --- | --- | --- | --- | --- | --- | --- | --- | --- | --- | --- | --- | --- | --- | --- | --- | --- | --- | --- | --- | --- | --- | --- | --- | --- | --- | --- | --- | --- | --- | --- | --- | --- | --- | --- | --- | --- | --- | --- | --- | --- | --- | --- | --- | --- | --- | --- | --- | --- | --- | --- | --- | --- | --- | --- | --- | --- | --- | --- | --- | --- | --- | --- | --- | --- | --- | --- | --- | --- | --- | --- | --- | --- | --- | --- | --- | --- | --- | --- | --- | --- | --- | --- | --- | --- | --- | --- | --- | --- | --- | --- | --- | --- | --- | --- | --- | --- | --- | --- | --- | --- | --- | --- | --- | --- | --- | --- | --- | --- | --- | --- | --- | --- | --- | --- | --- | --- | --- | --- | --- | --- | --- | --- | --- | --- | --- | --- | --- | --- | --- | --- | --- | --- | --- | --- | --- | --- | --- | --- | --- | --- | --- | --- | --- | --- | --- | --- | --- | --- | --- | --- | --- | --- | --- | --- | --- | --- | --- | --- | --- | --- | --- | --- | --- | --- | --- | --- | --- | --- | --- | --- | --- | --- | --- | --- | --- | --- | --- | --- | --- | --- | --- | --- | --- | --- | --- | --- | --- | --- | --- | --- | --- | --- | --- | --- | --- | --- | --- | --- | --- | --- | --- | --- | --- | --- | --- | --- | --- | --- | --- | --- | --- | --- | --- | --- | --- | --- | --- | --- | --- | --- | --- | --- | --- | --- | --- | --- | --- | --- | --- | --- | --- | --- | --- | --- | --- | --- | --- | --- | --- | --- | --- | --- | --- | --- | --- | --- | --- | --- | --- | --- | --- | --- | --- | --- | --- | --- | --- | --- | --- | --- | --- | --- | --- | --- | --- | --- | --- | --- | --- | --- | --- | --- | --- | --- | --- | --- | --- | --- | --- | --- | --- | --- | --- | --- | --- | --- | --- | --- | --- | --- | --- | --- | --- | --- | --- | --- | --- | --- | --- | --- | --- | --- | --- | --- |

Table A4. Logistic regression (unadjusted; dependent variable: onset of periodontitis) (N=337 unless specified otherwise)

**A4a Gender**

Logistic regression Number of obs = 337

LR chi2(1) = 3.25

Prob > chi2 = 0.0713

Log likelihood = -106.3799 Pseudo R2 = 0.0151

------------------------------------------------------------------------------

Ever_parod | Odds ratio Std. err. z P>|z| [95% conf. interval]

-------------+----------------------------------------------------------------

Gender |

Male | 1.943429 .721027 1.79 0.073 .9392177 4.021341

_cons | .0782123 .0217052 -9.18 0.000 .0453997 .1347402

------------------------------------------------------------------------------

Note: _cons estimates baseline odds.

**A4b Baseline Plaque index**

Logistic regression Number of obs = 337

LR chi2(1) = 5.67

Prob > chi2 = 0.0172

Log likelihood = -105.17124 Pseudo R2 = 0.0263

---------------------------------------------------------------------------------

Ever_parod | Odds ratio Std. err. z P>|z| [95% conf. interval]

----------------+----------------------------------------------------------------

Plaque_baseline | 1.018206 .0077963 2.36 0.018 1.00304 1.033601

_cons | .0444572 .0200256 -6.91 0.000 .0183874 .107489

---------------------------------------------------------------------------------

Note: _cons estimates baseline odds.

**A4c Baseline Marginal bleeding index**

Logistic regression Number of obs = 337

LR chi2(1) = 2.72

Prob > chi2 = 0.0991

Log likelihood = -106.64679 Pseudo R2 = 0.0126

------------------------------------------------------------------------------

Ever_parod | Odds ratio Std. err. z P>|z| [95% conf. interval]

-------------+----------------------------------------------------------------

MBI | 1.016284 .0100619 1.63 0.103 .9967536 1.036198

_cons | .0431622 .026582 -5.10 0.000 .0129087 .1443199

------------------------------------------------------------------------------

Note: _cons estimates baseline odds.

**A4d Baseline sites with PPD 4-5mm**

Logistic regression Number of obs = 337

LR chi2(1) = 6.11

Prob > chi2 = 0.0135

Log likelihood = -104.95375 Pseudo R2 = 0.0283

---------------------------------------------------------------------------------

Ever_parod | Odds ratio Std. err. z P>|z| [95% conf. interval]

----------------+----------------------------------------------------------------

sites PPD45 | 1.045813 .0179229 2.61 0.009 1.011268 1.081538

_cons | .0760929 .0186199 -10.53 0.000 .0471037 .122923

---------------------------------------------------------------------------------

Note: _cons estimates baseline odds.

**A4e Baseline teeth with PPD 4-5mm**

Logistic regression Number of obs = 337

LR chi2(1) = 5.34

Prob > chi2 = 0.0208

Log likelihood = -105.33672 Pseudo R2 = 0.0247

------------------------------------------------------------------------------

Ever_parod | Odds ratio Std. err. z P>|z| [95% conf. interval]

-------------+----------------------------------------------------------------

teethPPD45 | 1.081279 .0352259 2.40 0.016 1.014395 1.152572

_cons | .0705312 .0195592 -9.56 0.000 .0409573 .1214593

------------------------------------------------------------------------------

Note: _cons estimates baseline odds.

**A4f Baseline Smoking (≤10 cigarettes/day versus ≥11 cigarettes/day)** (N=293)

Logistic regression Number of obs = 293

LR chi2(1) = 5.36

Prob > chi2 = 0.0206

Log likelihood = -91.906281 Pseudo R2 = 0.0284

---------------------------------------------------------------------------------

Ever_parod | Odds ratio Std. err. z P>|z| [95% conf. interval]

----------------+----------------------------------------------------------------

1.Smoking_grt10 | 7.5 5.932311 2.55 0.011 1.591411 35.34599

_cons | .1 .0205688 -11.19 0.000 .0668217 .1496519

---------------------------------------------------------------------------------

Note: _cons estimates baseline odds.

**A4g Baseline Smoking (modified Pack-years)** (N=290)

Logistic regression Number of obs = 290

LR chi2(1) = 6.74

Prob > chi2 = 0.0095

Log likelihood = -90.906323 Pseudo R2 = 0.0357

------------------------------------------------------------------------------------

Ever_parod | Odds ratio Std. err. z P>|z| [95% conf. interval]

-------------------+----------------------------------------------------------------

adjusted_Pack_Year | 2.595513 .8778955 2.82 0.005 1.33757 5.036514

_cons | .0933165 .0202209 -10.95 0.000 .0610254 .1426943

------------------------------------------------------------------------------------

Note: _cons estimates baseline odds.

**A4h Baseline snuff use (yes/no)** (N=300)

Logistic regression Number of obs = 300

LR chi2(1) = 0.96

Prob > chi2 = 0.3268

Log likelihood = -94.828131 Pseudo R2 = 0.0050

------------------------------------------------------------------------------

Ever_parod | Odds ratio Std. err. z P>|z| [95% conf. interval]

-------------+----------------------------------------------------------------

1.snuff | 1.649824 .8106755 1.02 0.308 .6297697 4.322085

_cons | .0982906 .0214786 -10.62 0.000 .064048 .1508405

------------------------------------------------------------------------------

Note: _cons estimates baseline odds.

**A4i Baseline snuff use (modified Box-years)** (N=292)

Logistic regression Number of obs = 292

LR chi2(1) = 0.23

Prob > chi2 = 0.6326

Log likelihood = -89.881823 Pseudo R2 = 0.0013

-----------------------------------------------------------------------------------------

Ever_parod | Odds ratio Std. err. z P>|z| [95% conf. interval]

------------------------+----------------------------------------------------------------

adjusted_box_Year | .7242163 .5236108 -0.45 0.655 .1755686 2.987375

_cons | .1046546 .021815 -10.83 0.000 .0695548 .1574669

-----------------------------------------------------------------------------------------

Note: _cons estimates baseline odds.

Table A5. Logistic regression (adjusted for gender and modified pack-years; dependent variable: onset of periodontitis) (N=290)

**A5a Model 1 (Baseline Plaque index)**

Logistic regression Number of obs = 290

LR chi2(3) = 12.20

Prob > chi2 = 0.0067

Log likelihood = -88.174417 Pseudo R2 = 0.0647

------------------------------------------------------------------------------------

Ever_parod | Odds ratio Std. err. z P>|z| [95% conf. interval]

-------------------+----------------------------------------------------------------

Gender |

Male | 1.966775 .8401755 1.58 0.113 .8514018 4.543334

adjusted_Pack_Year | 2.524321 .9121614 2.56 0.010 1.243265 5.125374

Plaque_baseline | 1.010579 .0090431 1.18 0.240 .9930091 1.028459

_cons | .0395868 .0202975 -6.30 0.000 .0144914 .1081408

------------------------------------------------------------------------------------

Note: _cons estimates baseline odds

**A5b Model 2 (Baseline MBI)**

Logistic regression Number of obs = 290

LR chi2(3) = 11.98

Prob > chi2 = 0.0074

Log likelihood = -88.283512 Pseudo R2 = 0.0635

------------------------------------------------------------------------------------

Ever_parod | Odds ratio Std. err. z P>|z| [95% conf. interval]

-------------------+----------------------------------------------------------------

Gender |

Male | 2.076887 .8669764 1.75 0.080 .9164042 4.706941

adjusted_Pack_Year | 2.66808 .9435618 2.77 0.006 1.334053 5.33611

MBI | 1.012336 .0115434 1.08 0.282 .9899621 1.035215

_cons | .031197 .0227283 -4.76 0.000 .0074813 .1300912

------------------------------------------------------------------------------------

Note: _cons estimates baseline odds.

**A5c Model 3 (Baseline sites with PPD 4-5 mm)**

Logistic regression Number of obs = 290

LR chi2(3) = 14.44

Prob > chi2 = 0.0024

Log likelihood = -87.05272 Pseudo R2 = 0.0766

------------------------------------------------------------------------------------

Ever_parod | Odds ratio Std. err. z P>|z| [95% conf. interval]

-------------------+----------------------------------------------------------------

Gender |

Male | 1.957858 .820298 1.60 0.109 .8612856 4.450567

adjusted_Pack_Year | 2.56256 .9241264 2.61 0.009 1.263887 5.195647

sites PPD45 | 1.038475 .0197752 1.98 0.047 1.000431 1.077966

_cons | .0486314 .0174638 -8.42 0.000 .0240573 .0983076

------------------------------------------------------------------------------------

Note: _cons estimates baseline odds.

Table A6. Survival analysis for Periodontitis (≥2 teeth with PPD ≥6 mm; adjusted for gender, modified pack-years and number of sites with PPD 4-5 mm) (N=290)

Log likelihood = -42.708777 Number of obs = 290

------------------------------------------------------------------------------------

| exp(b) Std. err. z P>|z| [95% conf. interval]

-------------------+----------------------------------------------------------------

xb |

Gender |

Male | 1.994777 .7704261 1.79 0.074 .9357112 4.252524

adjusted_Pack_Year | 2.348341 .6758917 2.97 0.003 1.335892 4.128109

sites PPD45 | 1.035382 .0166654 2.16 0.031 1.003228 1.068567

_rcs1 | 2.523343 .4537902 5.15 0.000 1.773776 3.589663

_rcs2 | 1.402685 .2087443 2.27 0.023 1.04782 1.877734

_rcs3 | .9034854 .0661182 -1.39 0.165 .7827609 1.042829

_rcs4 | .967517 .0263385 -1.21 0.225 .9172476 1.020541

_cons | .0354431 .012032 -9.84 0.000 .018221 .0689432

------------------------------------------------------------------------------------

Note: Estimates are transformed only in the first equation.
